# Perspectives of people living with Parkinson's disease on personalized prediction models

**DOI:** 10.1111/hex.13500

**Published:** 2022-05-24

**Authors:** Lieneke van den Heuvel, Marjan Knippenberg, Bart Post, Marjan J. Meinders, Bastiaan R. Bloem, Anne M. Stiggelbout

**Affiliations:** ^1^ Department of Neurology, Centre of Expertise for Parkinson and Movement Disorders, Donders Institute for Brain, Cognition and Behaviour Radboud University Medical Centre Nijmegen The Netherlands; ^2^ Scientific Centre for Quality of Healthcare, Radboud Institute for Health Sciences Radboud University Medical Centre Nijmegen The Netherlands; ^3^ Scientific Centre for Quality of Healthcare, Centre of Expertise for Parkinson and Movement Disorders, Radboud Institute for Health Sciences Radboud University Medical Centre Nijmegen The Netherlands; ^4^ Department of Biomedical Data Sciences, Medical Decision Making Leiden University Medical Centre Leiden The Netherlands

**Keywords:** Parkinson's disease, patients' perspective, personalized prediction model

## Abstract

**Background:**

There is a great need for the development of personalized prediction models (PPMs) that can predict the rate of disease progression for persons with Parkinson's disease (PD), based on their individual characteristics. In this study, we aimed to clarify the perspective of persons diagnosed with PD on the value of such hypothetical PPMs.

**Methods:**

We organized four focus group discussions, each including five persons with PD who were diagnosed within the last 5 years. The sessions focused on what they think of receiving a personalized prediction; what outcomes are important to them; if and how the possibility of influencing the prognosis would change the way they think of personalized predictions; how they deal with the uncertainty from a PPM; and what barriers and facilitators they expect for using a PPM.

**Results:**

The wish of persons with PD for receiving personalized prognostic information was highly heterogenous, for various reasons. Most persons with PD would like to receive more personalized prognostic information, mainly to better prepare themselves and their loved ones for the future. The prediction provided should be as personalized as possible, and there should be adequate supervision and coaching by a professional when providing the information. They were particularly interested in receiving prognostic information when their interventions would be available that could subsequently influence the identified prognostic factor and thereby affect the disease course beneficially.

**Conclusion:**

Most persons with PD in this study want more insight into their own future by means of prediction models, provided that this is explained and supervized properly by professionals.

**Patient or Public Contribution:**

Two patient‐researchers were involved in the study design, conduct of the study, interpretation of the data and in preparation of the manuscript.

## 1 INTRODUCTION

In heterogeneous diseases, it is often difficult to determine an individually based prognosis. Parkinson's disease (PD) is a progressive, neurodegenerative disease with a fast‐rising prevalence worldwide.^1^ PD has a highly heterogeneous disease course, making it not (yet) possible to determine a reliable individual prognosis. Several studies have identified PD subtypes with a characteristic disease course, such as the diffuse malignant subtype with rapid disease progression, or the milder motor dominant subtype with an earlier age of onset and slower disease progression.^2^, ^3^ Even though these subtypes can be distinguished at the group level, they do not offer accurate representations of the individually expected disease course. There is evidence that some individual risk factors increase the risk of specific outcomes in PD. For example, higher age at diagnosis, male gender and severe axial disease symptoms are prognostic factors that are associated with a higher risk of mortality, while the presence of a cardiovascular risk profile is a risk factor for cognitive decline.^1^, ^4^, ^5^, ^6^, ^7^

Few models exist that attempt to make an individual prognosis by combining different risk factors for PD.^4^, ^8^ With newer, more advanced analysis techniques, which can process large amounts of data, we expect that the individual prognosis for many heterogeneous diseases, based on personal characteristics as well as disease characteristics, can be determined more accurately in the future.^9^ An example of such a personalized prediction model (PPM) is the U‐prevent tool.^10^ This tool calculates the risk of developing cardiovascular disease, based on individual characteristics such as age, gender, geographic region, body mass index, cholesterol level and blood pressure. The tool can also predict the number of healthy life years gained with an intervention, such as using statins.

Multiple studies have presented their ‘lessons learned’ when it comes to the use of PPMs in daily practice, for example by presenting barriers and facilitators perceived by physicians when using prediction models in practice.^11^ However, these studies generally focus on the professionals' point of view, but the perspective of the patient is typically lacking. More generally, there is a plethora of studies on how to communicate risks to patients, but there are relatively few studies on patient preferences for risk communication per se and their perspectives on what to communicate and what not. One study that did focus on the preference of chronic lung disease patients for predictive model characteristics, showed that patients were indeed highly interested in using prediction models as part of their care.^12^

Prediction models are expected to improve personalized care for patients, and therefore such models need to be designed around the preferences of the people they aim to serve. In the field of PD, the perspective of persons with PD on the use of PPMs in clinical practice has not been evaluated yet. It is known that at least some persons with PD would like to receive information on their prognosis, preferably early in the disease course.^13^ However, it remains unclear how persons with PD perceive the role of a PPM in their daily lives, what outcomes they value most, and how they deal with the uncertainties associated with predictions. In this study, we aim to gain a first insight into the perspectives of persons diagnosed with PD within the last 5 years on the use of PPMs in their daily lives. The chronic and highly heterogeneous nature of the disease makes PD a potential model condition for other chronic diseases.^14^ Findings from this study may therefore guide further research on the patients' perspectives on PPMs in other chronic diseases as well.

## 2 METHODS

### 2.1 Study design

We conducted qualitative research, using focus groups. We used focus groups as they offer a deep insight into the participants' views. Group discussion allows participants to consider answers that they normally would not have thought about themselves, providing a more complete and diverse set of responses compared to individual interviews.^15^ Our primary research question was: How do persons with PD perceive the role of a PPM in their daily lives? Our secondary research questions included: (1) What do persons with PD think of receiving a personalized prediction?; (2) What outcomes in prediction models are important to persons with PD?; (3) If the prediction can or cannot be influenced by an intervention, how does this change the way persons with PD think of a PPM?; (4) How do persons with PD deal with the uncertainty of prediction models and how certain should a prediction be before adding value for persons with PD?; and (5) What barriers and facilitators for using a PPM do persons with PD expect?

### 2.2 Population

We invited participants diagnosed with PD within the last 5 years, based on self‐report. To affirm the diagnosis, we aimed to include participants with a limited degree of functional disability associated with PD, because rapid disease progression within the first years after diagnosis is a red flag for PD.^1^ Practically, this meant that participants should have a normal balance. We operationalized a normal balance as the absence of frequent falls (≤1 in the past year) and a self‐reported score of ≤1 on item 2.12 of the Movement Disorders Society Unified Parkinson Disease Rating Scale (MDS‐UPDRS) (Over the past week, have you usually had problems with balance and walking?).^16^ We included participants without severe cognitive impairment, defined as a self‐reported score of 0, 1 or 2 on item 1.1 on the MDS‐UPDRS scale (Over the past week have you had problems remembering things, following conversations, paying attention, thinking clearly or finding your way around the house or in town?). No neurological examination was performed. Participants had to be able to speak Dutch. We aimed to include a maximum of five participants in each session, to ensure the discussion quality. We expected to reach data saturation after four focus groups sessions, however, additional sessions could be scheduled if data saturation would not be met.

### 2.3 Recruitment and consent

To recruit participants, an invitation was placed on the website and in the newsletter of the Dutch Parkinson's Disease Association.^17^ Potential participants could respond by phone or by e‐mail. If interested, they received an information letter containing more detailed information on the study, an informed consent form, and a short survey to check whether they fulfilled the inclusion criteria. This survey contained a question on the frequency of falls in the last year, items 1.1 and 2.12 of the MDS‐UPDRS scale, and demographical questions, including age, gender, and disease duration. Eligible participants were asked to return a signed informed consent form. We included the first 20 eligible participants, after which we started a waiting list. We manually assigned each participant to one of the four sessions, to ensure an equal distribution of gender and age across different sessions. M. K. and L. V. recruited the participants. There was no prior relationship between any of the researchers and the participants.

### 2.4 Procedure

**Figure 1:**
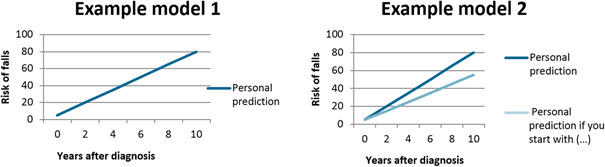
Fictitious examples of two simplified PPM's. Model 1 shows disease progression over time, and Model 2 shows disease progression over time, including how this would change when patients would adhere to a specific intervention. PPM, personalized prediction model

The topic guide was developed by the primary researcher (L. H.) and reviewed by the entire research group, together with two patient‐researchers (persons with PD who are trained to provide input in the study process, such as the study design and interpreting the results), and one additional patient who responded to the study invitation but was placed on the waiting list. The latter reviewed the questions in the topic guide in an online session with one of the researchers, to ensure clarity of the questions. The topic guide consisted of five parts to cover our research questions, and contained mostly open‐ended questions (the topic guide is provided in the Supporting Information). To address the question of the influenceability of the models, we showed the participants two fictitious, simplified, PPM's (Figure 1). Model 1 shows disease progression over time, while Model 2 shows disease progression over time, including how this would change when patients would adhere to an intervention (in the example: a more active lifestyle). To address uncertainty in prediction models, we asked participants what probability that something would happen to them should be provided by a prediction model for it to have added value. We presented an example of a small chance (1 person out of 100; 1%), 50‐50 (50 persons out of 100; 50%), or a high change of 95% (95 persons out of 100; 95%). The visual explanation that was used is presented in the topic guide in the Supporting Information.

The focus group sessions were conducted at the Radboudumc hospital in the Netherlands and were audio‐recorded. The sessions were moderated by L. H. (all sessions), and observed by M. K. (sessions 1–3) and L. V. (sessions 3 and 4). M. K. and L. V.  made fieldnotes during the sessions. At the beginning of each session, the moderator made it clear that participants could choose not to answer questions if they felt uncomfortable doing so. Participants were informed about the aim of the study, followed by a detailed explanation of a PPM (the Supporting Information). Group discussion was encouraged, and follow‐up questions were posed if clarification was needed. Each session was conducted in Dutch. Patients were reimbursed for travel and parking expenses and received a 50 euro gift card.

### 2.5 Coding and analysis

**Figure 2:**
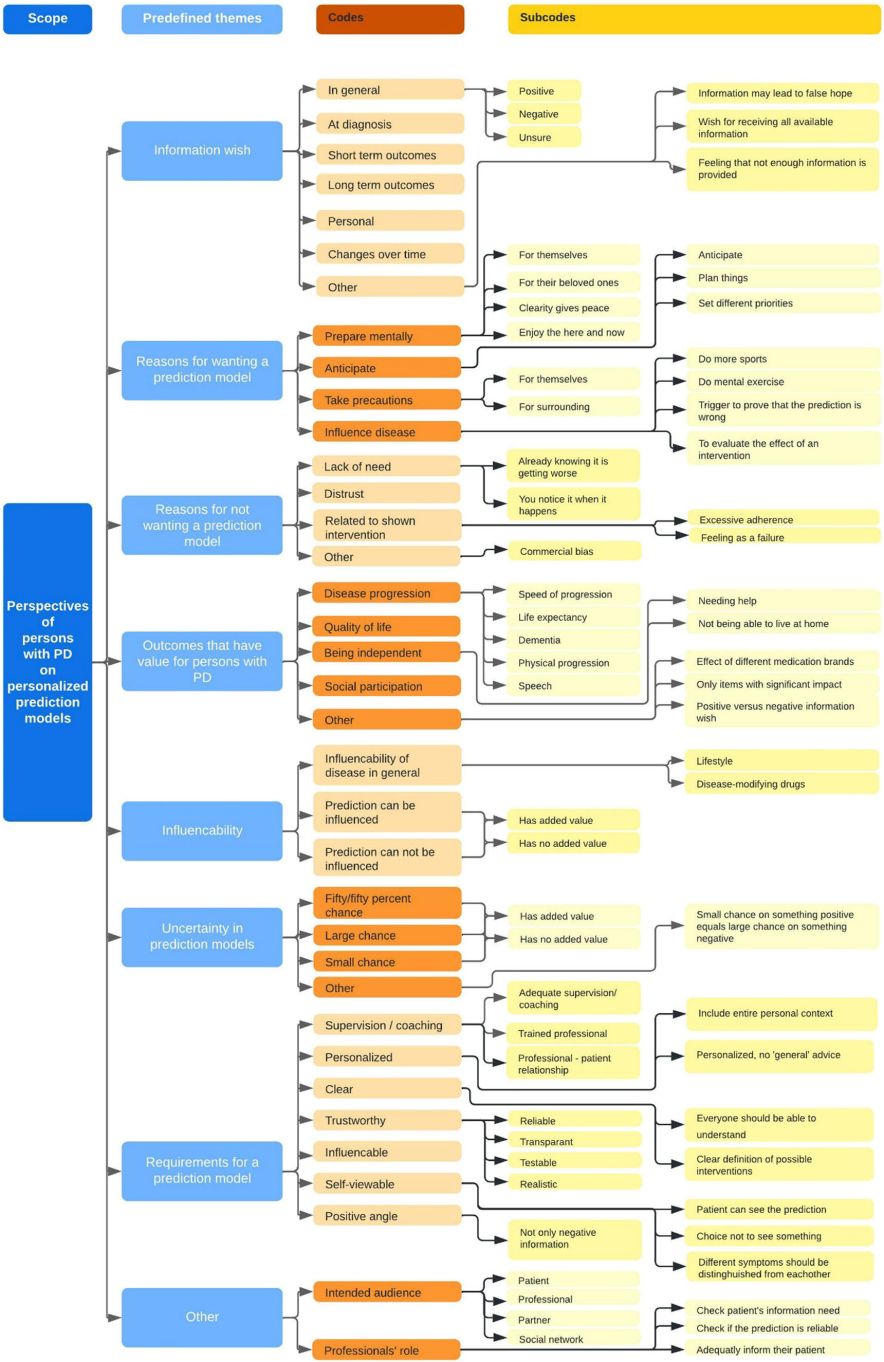
Description of the coding tree. PD, Parkinson's disease

The audio recordings were transcribed verbatim. The transcripts were not returned to the participants for correction. The Framework Method was used for thematic analysis of the transcripts.^18^ The thematic analysis enabled us to identify commonalities and differences in our data and to draw descriptive conclusions clustered around themes. The Framework Method consists of seven phases: (1) Transcription (2) Familiarizing with the data; (3) Coding; (4) Developing a working analytical framework (5) Applying the analytical framework; (6) Charting data into the framework matrix; (7) Interpreting the data.^18^ A deductive coding strategy was used, with some room for open coding, to enable potential other relevant themes to come up. Coding focused on items relevant to our research question, including what persons with PD think of receiving a personalized prediction; what outcomes are important to them; if and how the possibility of influencing the prognosis would change the way they think of personalized predictions; how they deal with the uncertainty from a PPM; and what barriers and facilitators they expect for using a PPM. A description of the coding tree is provided in Figure 2. We followed the recommendations outlined in the COREQ criteria as much as possible to analyze and report qualitative data (provided in the Supporting Information).^19^ Though data analysis was performed after completion of the data collection, an additional focus group could be organized if the analysis would show that data saturation was not met. Data saturation was defined as the point at which no new information was derived from the focus group. Coding of the first transcript was performed independently by two researchers (L. H. and M. K.), using ATLAS.ti 8.4.20 software. Differences in coding were resolved by discussion and the researchers agreed on an initial coding scheme. The second and fourth transcripts were coded by L. H. and checked by M. K., and the third transcript was coded by M. K. and checked by L. H. Differences in coding were resolved by discussion and the coding scheme was adapted accordingly after each transcript. Finally, L. H. reviewed all transcripts for consistency in coding. The results were discussed with all members of the research team, including the patient‐researchers. The latter helped put the results in a patient‐derived perspective.

### 2.6 Ethical statement

The study protocol was approved by the Medical Ethics Committee of the Radboud university medical center and registered as 2021‐13037. All participants gave written informed consent before the focus group discussions.

## 3 RESULTS

In total, 32 individuals responded to the study invitation. The first 20 individuals all met the inclusion criteria and were included in the study. After inclusion, and a few days before the first session, one participant dropped‐out of the study due to practical reasons. We replaced this participant with the first participant on the waiting list who was available on that date. The included participants were divided into four focus group sessions with five participants in each session (Table 1). Data saturation was reached after three focus group sessions, which was confirmed in the fourth session. The sessions lasted for 65–80 min.

**Table 1:** Demographic and clinical characteristics of the participants in the focus group discussions

	All participants	Session 1	Session 2	Session 3	Session 4
Number	**20**	5	5	5	5
Age (years [SD])	**66 (9)**	68 (7)	62 (10)	67 (8)	66 (13)
Gender (*n* [%] men)	**11 (55%)**	4 (80%)	2 (40%)	2 (40%)	3 (60%)
Months since diagnosis (mean [SD])	**23 (17)**	31 (15)	18 (17)	29 (21)	14 (8)
MDS‐UPDRS item 1.1^a^					
Score 0 (*n*)	**13**	**3**	**2**	**5**	**3**
Score 1 (*n*)	**3**	**0**	**1**	**0**	**2**
Score 2 (*n*)	**4**	**2**	**2**	**0**	**0**
MDS‐UPDRS item 2.12^b^					
Score 0 (*n*)	**13**	**4**	**3**	**3**	**3**
Score 1 (*n*)	**7**	**1**	**2**	**2**	**2**

Abbreviation: SD, standard deviation.

^a^ MDS‐UPDRS item 1.1: ‘Over the past week have you had problems remembering things, following conversations, paying attention, thinking clearly or finding your way around the house or in town?’. Answer options include: 0 = Normal; 1 = Slight; 2 = Mild; 3 = Moderate; 4 = Severe.

^b^ MDS‐UPDRS item 2.12: ‘Over the past week, have you usually had problems with balance and walking?’. Answer options include: 0 = Normal; 1 = Slight; 2 = Mild; 3 = Moderate; 4 = Severe.

### 3.1 Patient's wish for receiving prognostic information

Most participants had a positive information wish, for example, they would like to receive more information on their individual prognosis. The most important reason for having a positive information wish was to prepare themselves (e.g., to prepare themselves mentally, to anticipate, to take concrete precautions and/or to prepare their loved ones). A specific, spontaneously mentioned reason, was the potential to influence the course of the disease, or to prevent the prediction from becoming true, by acting now.

Some participants preferred only a little or no information about their individual prognosis, mainly due to their perception that knowing a (negative) prediction could influence the quality of life now, for example, by causing depression and fear, or by feeling forced to enjoy themselves in the ‘good’ time they have left. A few participants mentioned a lack of need for a personalized prediction (e.g., because you will notice it when it happens; or because you already know that it is getting less) or distrust in the underlying model (i.e., disbelief that it is possible to make an individual prediction).

Some participants explicitly indicated that they were unsure about how much information they would prefer. The wish for receiving information on individual prognosis could differ for different outcomes and could change over time. For example, participants had a variable information wish for prognosis regarding mobility or dementia. Some participants preferred to have only a prediction for short‐term outcomes and not long‐term, while other participants felt the other way around.

Several participants indicated that they felt as if they were left to themselves when it came to finding information on the impact of the disease on their lives. They would like to have (had) more information, preferably early after the initial diagnosis, on what the disease and prospects of their disease implied to them individually.

Some participants specifically highlighted the potential risks of PPMs, including the risk of commercial use of prediction models. They were afraid that pharmaceutical companies would use the predictions to advertise treatments, which, they thought, would make the prediction less objective.

Tables 2 and 3 provide an additional explanation of the themes described above, supported by quotations from the participants.

**Table 2:** Patients wish for receiving individual information on prognosis

Theme	Explanation	*Examples*
Positive information wish	Most participants had a positive information wish, for example, they would like to receive more information on their individual prognosis. This information wish could differ for different outcomes.	‘Here you notice that everyone is in it in a completely different way. So, yes, keep the information coming. I'll see what I do with it’. (FG3, SP6)
Negative information wish	Some participants preferred only little or no information about their individual prognosis.	‘Yes, I like just not knowing some things.’ (FG2, SP6)
Unsure information wish	Some participants explicitly indicated that they were unsure about how much information they would prefer.	‘On the one hand I want to know so that I can influence it; on the other hand, I don't really want to concern myself with it because we all have to live in the here and now because you don't know what will happen tomorrow anyway’. (FG3, SP4)
Information wish is personal	The information wish varied across individuals and outcome measure (e.g., different information wish for prognosis regarding mobility or dementia).	*‘*I think it's useful that you always ask the person themselves, “What do you want to know?” Hey, so if someone says, “I don't want to know”, it's obviously not helpful to say, “Yeah, it's going to happen like this”’. (FG4, SP5)
Timing of receiving information	Several participants indicated that they would have wanted more information early after diagnosis on (1) what their disease means for them as individuals and (2) what their individual prognosis is. Some participants felt that they were on their own when it comes to finding this information.	‘So, I really missed someone to ask: “Well, what's going to happen to me now? What can I still do and what can I continue to do? Can I still control myself?” I missed all that. I still miss that now. I don't know what's happening to me. I really miss that’. (FG4, SP6)
Short‐term future versus long‐term future	Some participants preferred to have only a prediction for short‐term outcomes and not long term, and other participants the other way around. Participants used varying definitions for short term and long term.	‘I don't care where I am in five years. I am interested in where I will be next year. <> Well, you probably know that expression, “We cross that bridge when we get there”. <> I don't know where I'll be in five years. But I do want to be prepared for next year, because I can do something about that. In five years I can't do anything about it’. (FG1, SP5)
Information wish changeable over time	One participant mentioned that his information wish changed over time (i.e., he used to have a negative wish for information but this changed to a positive wish for information due to disease acceptance).	‘Five years ago, if you'd have asked me the same question, I wouldn't need to know anything. <> So now I changed to the other side. <> It starts with accepting the fact that you have it’. (FG4, SP4)

**Table 3:** Patients perspective on receiving a personalized prognostic prediction

Theme	Explanation	*Examples*
*Reasons for wanting a personalized prognostic prediction*		
Prepare mentally	Feeling prepared, clarity gives peace, knowing the future makes is easier to enjoy now	‘Well, then you are prepared for it. And not only me, but also my partner and my children. So, that… Yes. I want to know everything’. (FG3, SP3)
Anticipate	Plan things, change priorities, change daily schedule	‘Then you can perhaps anticipate, whether you can do something with it, or whether you can prepare something’. (FG2, SP5)
Take concrete precautions	Change living or working situation (e.g., move to apartment, sell company), get aids (e.g., wheelchair), advanced care planning (e.g., prepare statement of will)	‘Well, I want to know until what point I can function as I function now. And if that is no longer possible then I have to take measures. I have to sell my company, give my dog an address, et cetera, et cetera’. (FG4, SP4)
Prepare loved ones	Mentally prepare their loved ones, taking concrete precautions for people surrounding them	‘I would love to know, if only for your social environment and family, to prepare them for that’. (FG3, SP3)
Influencing disease by taking action now	Being able to prevent prediction to come true (e.g., by improving physical activity or perform mental exercises)	‘If there is a prediction model then I have a better, in my opinion, a better set of instruments to determine and implement my approach. To see if maybe I don't want to end at the left, but at the right’. (FG1, SP3)
*Reasons not to want a personalized prognostic prediction*		
Knowing could influence quality of life now	Risk on depression or fear when knowing a (negative) prediction. Hyperfocus on the future disrupts living in the present. Feeling forced to enjoy the present	‘And, such a prediction can of course work against you, it can make you a bit more depressed than if you didn't know anything’. (FG2, SP3)
Lack of need	You notice it when it happens. You already know that it is getting less	‘We all know it's decreasing. <> that doesn't have to happen yet, but yes, you anticipate anyway. So yeah, I don't need a model for that’. (FG3, SP4)
Distrust	Distrust in the underlying model. Disbelief that it is possible to make an individual prediction	‘Not yet, but that also has to do with the fact that I don't have a lot of confidence in what a prediction model can ultimately deliver’. (FG3, SP5)
*Specific risks of a personalized prediction model*		
Risk of commercial bias	Risk of too much influence from pharmaceutical companies	‘Before you know it, there are advertisements from some pharmaceutical company saying: take this pill and you won't get that again’. (FG1, SP5)
Risks associated with showing the effect of an intervention	Risk of going too far in adhering to an interventionRisk of feeling as a failure if they fail to influence their disease as much as the model had predicted	‘When they say, “That helps”, I would really go wild, all the way through everything, all the pain and everything’. (FG3, SP4) *‘*And if you can't meet its expectations, then I would start having all kinds of thoughts about it again, which would get in the way again’. (FG4, SP2)

### 3.2 Outcomes that persons with PD value most

Participants with positive information wish to mention a range of outcomes for the PPM. Outcomes mentioned covered the following themes: disease progression in general (e.g., how fast does my disease progress?), progression in physical (e.g., how is my ability to be physically active progressing over time?) and mental functioning (e.g., do I get dementia? And when?), being independent (e.g., when do I need help? When can I not live at home anymore?), social participation (e.g., how long can I actively participate in my social life?), and quality of life (e.g., how long do I have a good quality of life?). Mental functioning was the single outcome, mentioned by several participants with a negative information wish. Though they felt no need to receive predictive information, they would like to receive information on dementia, to have a possibility to act before they would lose self‐control (i.e., being able to make end‐of‐life decisions).

### 3.3 Patient's perspective on being able to influence a personalized prediction

When asked, almost all participants agreed that they would like to have more information on what they could do to influence the course of their disease. When showing the participants the two fictitious, simplified PPM's as presented in Figure 1, most participants said that they would like to see both models, in agreement with their positive wish for information. However, some participants who at first indicated that they would not want to see a prediction of their disease, changed their opinion when a prediction would also show the effect of an intervention.

Some participants mentioned a potential risk of seeing the effect of an intervention in a PPM. They were afraid of going too far in adhering to the intervention (e.g., excessive exercise in the example of a more active lifestyle), and the risk of feelings of failure if they would not influence their disease as much as the model had predicted. They stressed that any intervention shown to influence the disease needs to be well defined and proper supervision and coaching by a professional is needed.

### 3.4 Handling uncertainty in predictions

In general, it was difficult for participants to talk about uncertainty in hypothetical predictions, when we presented examples with varying levels of uncertainty. Most participants understood that a prediction does not equal certainty (e.g., the prediction doesn't come true, by definition, it is a direction). Participants differed in their reaction to uncertainty, ranging from hoping for the best or fearing the worst, to being ambivalent. Most participants agreed that, if they could choose, they would favour a prediction with the lowest level of uncertainty (i.e., a very large, or a very small chance that the prediction will come true, as the 50% represents a lottery, or the general picture, which you also can find on the internet).

### 3.5 A patient's ‘wish list’ for PPMs

Participants mentioned several requirements for a PPM, which we summarized as a patients' ‘wish list’ for PPM's (Table 4). Adequate supervision and coaching by a professional, for example, a neurologist or Parkinson's nurse, was mentioned by most participants as an important requirement for using a PPM. The professional should be able to adequately elicit the individual's information wish. Also, the professional should be skilled in risk communication and able to judge to what extent the prediction would be applicable to an individual patient. Other important requirements for a PPM were that the prediction should include the entire patient's perspective (i.e., being ‘truly’ personalized); it should be trustworthy (i.e., trustworthy, realistic, and transparent about what the prediction is based on); self‐viewable for patients including an option not to see something; influenceable; understandable; and/or have a positive angle (i.e., it should not just be negative).

**Table 4:** A patients' ‘wish list’ for personalized prognostic prediction models

Theme	Explanation	Examples
Adequate supervision and coaching	A professional should elicit the individual's wish for information. The professional should be able to judge the trustworthiness of the prediction. Predictions should be discussed by a professional and adequate coaching should be provided.	‘The doctor must also be able to communicate about this’. (FG3, SP5) ‘It has to be translated to the situation where you are’. (FG3, SP6) ‘There should be aftercare, and intermediate care. <> total care’. (FG1, SP5)
Personalized	The entire patient's perspective should be included in the prediction model. No ‘general’ advice.	*‘*What should not be in it for me is the general well‐intentioned advice: “Keep breathing. Keep moving. Good nutrition”. (FG1, SP3) ‘That you notice that it is aimed at you personally’. (FG4, SP2)
Trustworthy	The model should be trustworthy, realistic and it should be transparent what the prediction is based on.	‘It must be reliable and it must be transparent. They have to be able to see how the process went to see how the algorithm came to a decision. That must be traceable. And the question is whether that is possible’. (FG3, SP5)
Self‐viewable	The model must be self‐viewable for some participants. There should be a choice to not see something (e.g., see only outcomes that have a large impact for them as individual).	*It would be nice if you could look it up and that you could also formulate your questions in advance before you go for an appointment with the neurologist*.(FG2, SP4)
Influenceable	For some participants, the model should give them the opportunity to do something about it/to avoid the prediction from coming true. They suggested that feedback should be provided to see if they are on the right track.	‘Those prediction models are fine, only if you could do something about it now’. (FG1, SP2) ‘Halfway through I can see what happens if I continue to live like this, then I know. But if I improved myself? How does it change?’. (FG4, SP6)
Clear	Everyone should be able to understand the prediction model. If a possible intervention is shown, that intervention must be clearly defined.	‘The model must be applied in such a way that people can deal with it, in the broadest sense of the word – whatever your background’. (FG3, SP5)
Positive angle	It shouldn't just be negative.	‘So in a prediction model there shouldn't be only negative things, like: Yeah, this is what's in store for you and in five years you'll be absolutely dead’. (FG1, SP5)

## 4 DISCUSSION

In this study, we gained a more detailed insight into the perspectives of persons diagnosed with PD within the last 5 years on the use of PPMs in daily clinical practice. We found that the wish of persons with PD for receiving personalized prognostic information is highly heterogenous. Most persons with PD would like to receive more personalized prognostic information, mainly to better prepare themselves and their loved ones for the future. That the majority of people who want to receive information on their prognosis is seen outside of PD as well, for example in persons suffering from chronic lung disease,^12^ or persons with traumatic spine injury.^20^ We found that the extent to which persons with PD want to receive information differs between individuals can change over time, and might sometimes be contradictory. These findings align with findings on advance care planning (ACP) in PD. ACP is a process that supports individuals in understanding and sharing their values, life goals and preferences regarding future medical care.^21^ Persons with PD and caregivers describe a variety of different personal views on ACP, shaped by their own personal values, and each personal view influences the individual's willingness to discuss ACP.^22^ Persons with PD in our study also indicated that a PPM might be just as important for informing caregivers, as it is for informing themselves, for example, to prepare their caregivers for what is coming. The importance of including the caregivers' perspective has been underlined in ACP as well.^22^

For many persons with PD, it is important that a prediction provided by a PPM can be influenced, for example by lifestyle changes or a putative disease‐modifying treatment. This can be understood by the notion that when being able to influence the prognosis, provides a feeling of being in control. However, even though several promising disease‐modifying treatments are being tested in (pre‐)clinical trials, it is currently not possible to slow down disease progression in PD. Even when disease‐modifying treatments become available, it might still take years before we are able to predict the effect of such interventions at an *N* = 1 level. Showing the expected effect of disease‐modifying interventions at an individual level carries the risk of people losing themselves in adhering to an intervention or feeling like a failure when they fail to comply. Therefore, if a PPM, showing the individual expected effect of disease‐modifying interventions, would become available, it should be clear how interventions are defined and what persons with PD can expect. It is important to notice that the decision to start disease‐modifying treatment is not purely evidence‐based. For example, a study in multiple sclerosis that focused on the patients' perspective found that the decision to start disease‐modifying treatment involves themes such as dealing with the constant confrontation with the disease, managing inevitable decline and the hope of delaying disease progression.^23^

Because PPMs ultimately aim to serve the persons with the disease and their clinicians, their needs should be leading when developing such models. In our study, we present requirements that PPMs should meet before having added value for persons with PD. First, persons with PD indicate that PPMs are specifically useful when they are truly personalized, meaning that a prediction for an individual is based on his or her individual personal characteristics as well as disease characteristics as much as possible. However, PD is a complex disease, and direct and indirect effects of individual (disease‐) characteristics on the disease course are not always clear.^1^ This complicates the development of PPMs. Furthermore, an important requirement identified by persons with PD for using PPMs is adequate supervision and coaching by a professional. This professional should be trained in elucidating the individual's information wish and should be equipped to communicate the impact and limitations of a PPM. Especially dealing with the uncertainty that inevitably comes with any prediction model can be challenging and stressful. Persons with PD are vulnerable to the negative effects of psychological distress, and (motor) symptoms, for example, can worsen in stressful situations.^24^ Some of the requirements of a PPM from the patients' perspective overlap with requirements emphasized by health care professionals, mainly regarding the accuracy of the models, clarity, and ease of use.^11^, ^25^, ^26^

The use of a PPM has important ethical considerations. Concerns include liability in cases of medical error, doctors' understanding of how tools produce predictions, patients' understanding and control of how tools are used in their care, and issues around privacy, security, and control of patient data.^27^ In our study, some participants mentioned the risk of commercial use of the models, for example in pharmaceutical advertisements, which, they think, would make PPMs less objective. The importance of privacy issues and control of patient data was emphasized by our patient‐researchers, and they raised concerns about the pressure that, for example, employers or insurance companies can put on the patient to provide information.

The major strength of this study is that we are the first who evaluated the perspective of persons with PD on PPMs. Following the increasing number of studies that focus on the development of PPMs, we expect that such models will become available in the future. With its progressive and heterogeneous disease course, PD is a potential model condition for other chronic (neurological) diseases as well.^14^ In our study, we do not only present the perspective of persons with PD on such models, but we also present requirements that PPMs should meet before having added value for persons with PD. The results of this study might be applicable to other chronic diseases as well, even though this should be established by further research. Also, both in the study design and in interpreting the results, we cooperated with two patient‐researchers. Their experience with the disease helped us with putting the study results in a patient‐derived perspective.

This study is not without limitations. First, as actual PPMs have not been operationalized in a clinical setting yet, this study has a hypothetical character. It might be difficult for persons with PD to imagine what a PPM would look like and what impact it might have on them. However, the hypothetical character allowed participants to provide a broad view of all kinds of implications and outcomes from PPMs. Second, we realize that the way we asked our questions, could potentially prime other participants into answering in a certain direction, which might bias the results. However, we tried to keep the questions as neutral as possible, and we challenged participants to provide answers from their own unique perspectives. Third, we used a mainly deductive thematic analysis method, based on our predefined research questions, which might have caused that we missed themes. However, we did leave room for open coding in the analysis to enable potential other relevant themes to come up and minimize the risk of missing themes in the analysis that are important for persons with PD. Fourth, the mean disease duration in focus group sessions one and three was slightly longer compared to sessions two and four. We found that someone's wish for information can change over time, and it is possible that disease duration also influences individual preferences. Even though we found no large differences in outcomes between different subgroups, the relatively small sample size makes it difficult to accurately compare opinions between individuals with different disease durations. Fifth, our inclusion criteria came with some limitations. We included persons diagnosed with PD within the last 5 years, and we consequently missed persons who were in an advanced disease stage. The opinion of persons who are in more advanced disease stages might differ from the study population included in this study, for example, because values and goals might change over time. Therefore, results cannot automatically be extrapolated to different disease stages. Also, the participants in our study were selected based on a self‐reported diagnosis, and therefore we cannot provide absolute certainty that our participants were diagnosed with PD. Furthermore, while we only included persons with PD, participants mentioned the important role of caregivers. Future studies should therefore include the perspective of caregivers on PPMs as well. Finally, we included participants from the Netherlands only. Persons with PD from different countries might have a different view on the use of PPMs, for example due to cultural differences. Therefore, our study needs validation in other countries or cultures.

In conclusion, most persons with PD in this study do want to gain insight into their own future by means of prediction models, when this is properly explained by professionals. Patients were particularly interested in receiving prognostic information when there are interventions available that can subsequently influence the identified prognostic factor and thereby affect the disease course beneficially.

## AUTHOR CONTRIBUTIONS

Lieneke van den Heuvel, Bart Post, Marjan J. Meinders, Anne M. Stiggelbout and Bastiaan R. Bloem were involved in conceptualizing the study. Lieneke van den Heuvel and Marjan Knippenberg collected and analyzed the data. Lieneke van den Heuvel prepared the original draft of the manuscript. All authors reviewed and revised the manuscript for intellectual content.

## CONFLICTS OF INTEREST

The authors declare no conflicts of interest.

## Supporting information

Supporting information.

## Data Availability

The data that support the findings of this study are available on request from the corresponding author. The data are not publicly available due to privacy or ethical restrictions.
