# Supplementary material for: Perspectives of people living with Parkinson's disease on personalized prediction models
Source: Health Expect. 2022 May 24;25(4):1580–90. doi: 10.1111/hex.13500 (PMC9327833; doi:10.1111/hex.13500)
Supplement: Supplementary file 1 — Supporting information. [file HEX-25--s001.docx]

Supplementary material

# Supplement 1. COREQ checklist

**Domain 1: Research team and reflexivity**

Personal characteristics

1. Interviewer/facilitator: Methods, *procedure* and *coding and analysis section*. Page 4-5.
2. Credentials: Title page.
3. Occupation: Title page.
4. Gender: Title page.
5. Experience and training: Title page.

Relationship with participants

1. Relationship established: Methods, recruitment and consent section. Page 4.
2. Participant knowledge of the interviewer: Methods, recruitment and consent section. Page 4.
3. Interviewer characteristics: Methods, recruitment and consent section. Page 4.

**Domain 2: Study design**

Theoretical framework

1. Methodological orientation and Theory: Methods, coding and analysis section. Page 5.

Participant selection

1. Sampling: Methods, *population* and *recruitment and consent* section. Page 3-4.
2. Method of approach: Methods, *recruitment* *and* *consent* section. Page 4.
3. Sample size: Methods, *population* section. Page 3.
4. Non-participation: Results, first paragraph. Page 6.

Setting

1. Setting of data collection: Methods, *procedure* section. Page 4-5.
2. Presence of non-participants: Methods, *procedure* section. Page 4-5.
3. Description of sample: Results, first paragraph. Page 6. Table 1.

Data collection

1. Interview guide: Methods, *procedure* section. Page 4.
2. Repeat interviews: Not applicable.
3. Audio/visual recording: Methods, *procedure* section. Page 5.
4. Field notes: Methods, *procedure* section. Page 5.
5. Duration: Results, first paragraph. Page 6.
6. Data saturation: Results, first paragraph. Page 6.
7. Transcripts returned: Methods, *coding and analysis* section. Page 5.

**Domain 3: Analysis and findings**

Data analysis

1. Number of data coders: Methods, *coding and analysis* section. Page 5.
2. Description of the coding tree: Provided in the supplement.
3. Derivation of themes: Methods, *coding and analysis* section. Page 5.
4. Software: Methods, *coding and analysis* section. Page 5.
5. Participant checking: Methods, *coding and analysis* section. Page 5.

Reporting

1. Quotations presented: Table 2-4
2. Data and findings consistent: There is consistency between the data presented in the Results section and the study findings. Interpretations and theories generated are presented in the Discussion section of the main manuscript.
3. Clarity of major themes: Major themes are presented in the main manuscript.
4. Clarity of minor themes: Some minor themes are presented in the main manuscript.

# Supplement 2. Interview guide

**Explanation of prediction models**

It is still very difficult to predict how Parkinson's disease will behave over time for individual patients. To improve this, so-called prediction models are currently being developed. To do this, a mathematical formula uses the medical data of large groups of people with Parkinson's disease to predict how the disease will behave in an individual with Parkinson's disease. For example, an attempt is made to predict as precisely as possible how someone's illness will progress and when someone will develop certain complaints, on the basis of a person's context and disease characteristics.

Perhaps such a prediction model will show that, for example, a person for whom we enter data, such as gender and age, into the model has a high chance to fall 5 years after the diagnosis. If we enter the data from a completely different person, this might be only after 10 years. This is a made-up simple example, but it does show the possibilities of a prediction model. The real prediction models will be more complicated and will include much more data, such as age, gender, which complaints they have, and other diseases someone has. The more characteristics that are put in the prediction model, the better the prediction fits that person. The outcome is a prediction, not an assurance that it will happen. A prediction model can never provide complete certainty.

**Interview questions**

Part 1) General opinion of PwPD on receiving a personalized prediction on their prognosis

Suppose a prediction model shows you which Parkinson's complaints you will get in 5 years or in 10 years, would you like to know this information? Why or why not? Or would you like only your neurologist to know this information but not necessarily discuss it with you?

Part 2) What outcome measures do PwPD want?

If you have a prediction model for the course of your disease, what would you like to have predicted and why?

Part 3) If the prognosis can or cannot be influenced, how does this change how PwPD think of a prognostic prediction model?

Example:
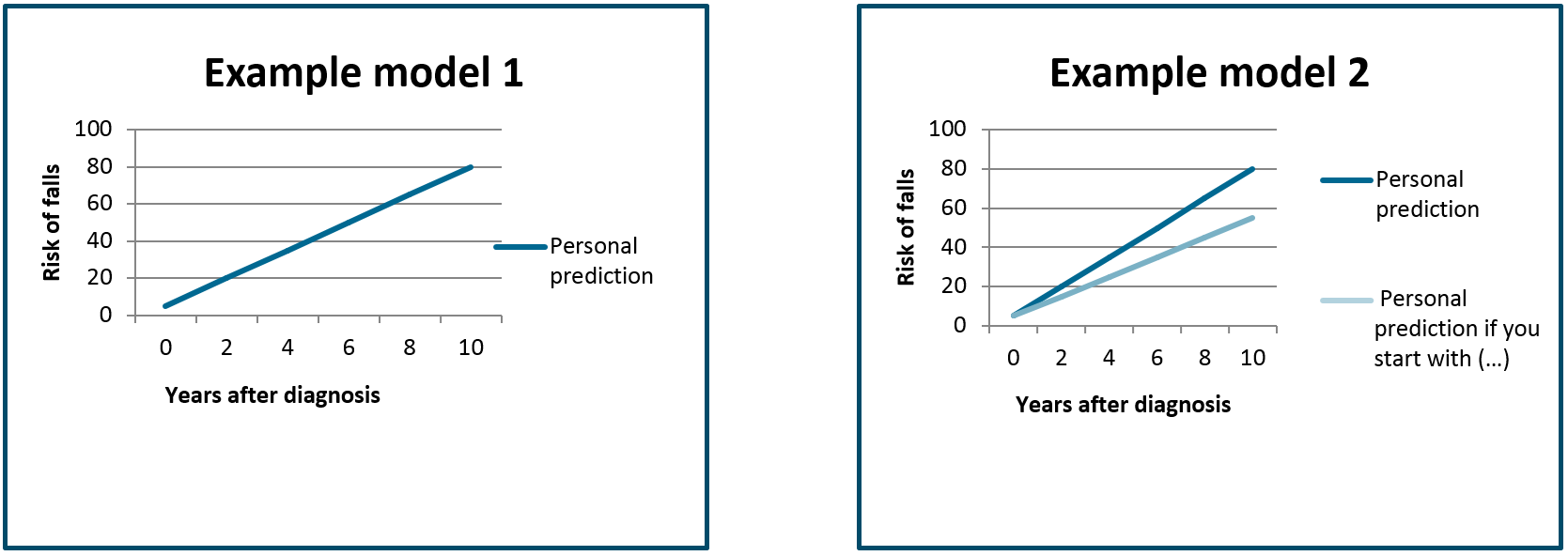


This picture shows two made-up examples of prediction models. The first one is the left picture. Here you can see how the risk of falling increases for the person for whom this prediction is made the longer the disease exists. For example, if someone has the diagnosis for 10 years, there is an 80% chance of experiencing falls. In the second picture you see the same prediction, but there is also a line that indicates what the chance would be for that person IF he or she starts with an intervention. This intervention could be anything, for example living healthier from now on. This picture 2 shows how things will go if the person continues like this, but also what that person can do to change this. This is a made-up example for the risk of falling, but it could also be about other things that are important to you.

Which prediction model has added value for you and why? Only 1, only 2, or both?

Part 4) What certainty should a prediction model provide before the model has added value for PwPD?

A prediction model cannot assure complete certainty that something will happen. To explain this, we want to give the weather forecast as an example. The weather forecast tries to predict whether it will rain that day, and if so at what time. When the forecast, for example, says that there is a 10% chance of rain that day, that means it will probably stay dry, but this is not certain. That's how it works with a prediction model. The model can predict the chance that you will develop certain complaints in a certain number of years, but this is not completely certain and it is always possible that it is different in your case.

A prediction model can tell you, for example, whether the chance that you will suffer from something is high, small, or fifty-fifty. Then the prediction model says, for example, that the chance that you will suffer from a certain complaint in 5 years is 95%. This means that in 5 years 95 out of 100 people will be affected by that complaint, but also that 5 out of 100 people will not be affected by it. An example of a small chance is 1%, where in 5 years only 1 person out of 100 people will suffer from that complaint and 99 people will not.

*Example:*


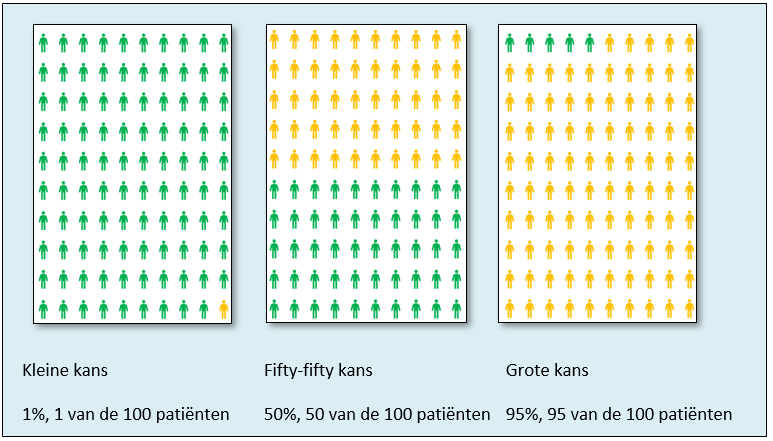


In addition to the uncertainty in the prediction, the prediction may be something that is good for you (e.g. that the disease progresses very slowly or that you do not develop dementia) or something that is not good for you (e.g. that the disease deteriorates very quickly, or that you become dependent on a wheelchair, or suffer from dementia). What probability that something is going to happen for you should a prediction model provide before it has added value for you to know? Can you explain why? Does it matter to you whether it is something that is good for you or something that is not good for you?

Part 5) Barriers and facilitators for using prediction models.

Suppose your neurologist would like to use a prediction model in the doctor's office to start a conversation about the future with you. What requirements does this prediction model have to meet for you to want to use it? Which things make it more pleasant or more unpleasant for you to use a prediction model?
